# Supplementary material for: Morphology and synapse topography optimize linear encoding of synapse numbers in Drosophila looming responsive descending neurons
Source: bioRxiv. 2024 Apr 28:2024.04.24.591016. Preprint. [Version 1] doi: 10.1101/2024.04.24.591016 (PMC11071487; doi:10.1101/2024.04.24.591016)
Supplement: Supplement 1 [file NIHPP2024.04.24.591016v1-supplement-1.pdf]

bioRxiv preprint doi: https://doi.org/10.1101/2024.04.24.591016; this version posted April 28, 2024. The copyright holder for this preprint (which was not certified by peer review) is the author/funder, who has granted bioRxiv a license to display the preprint in perpetuity. It is made available under aCC-BY-NC-ND 4.0 International license.

SUPPLEMENTAL FIGURES

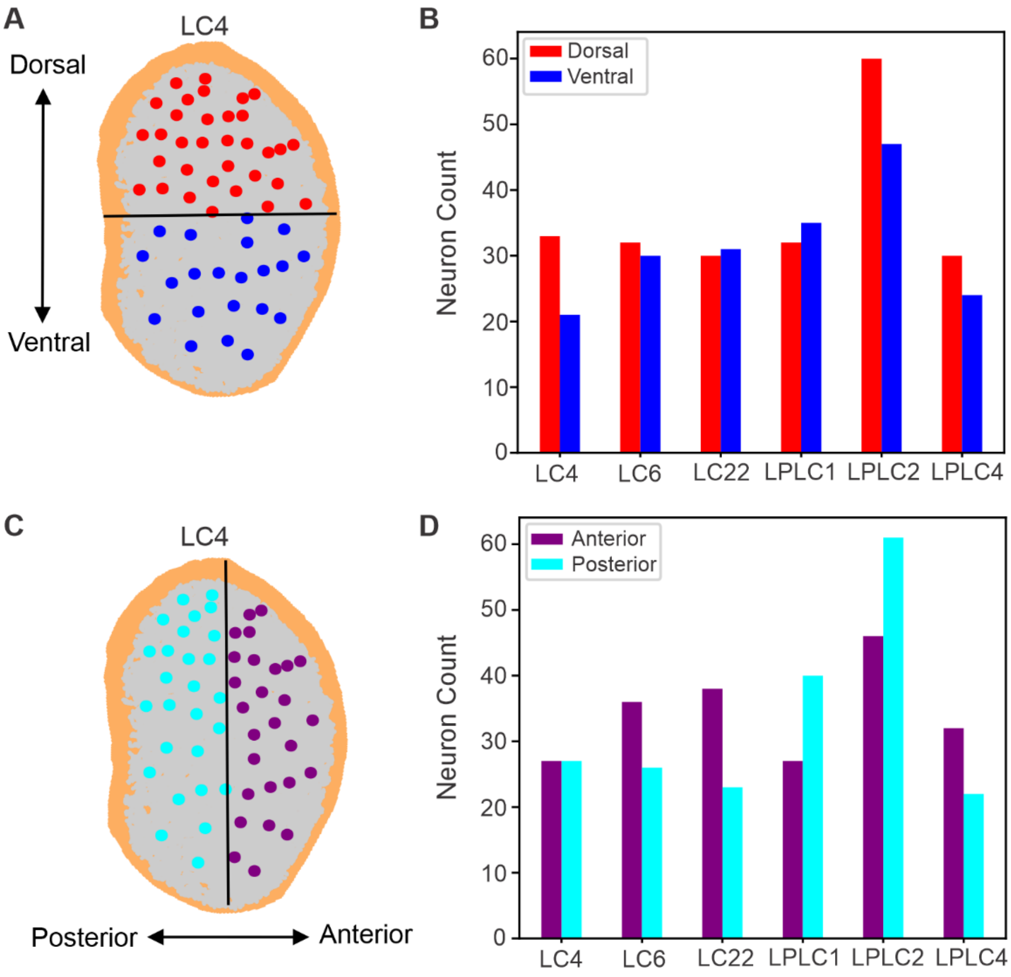

Figure 3—figure supplement 1. VPN centroids tile the lobula with biases across the two main axes. (**A**) Example LC4 centroid (dorsal: red, ventral: blue) and their dendrite (gray) projections onto the lobula. The black line splits the lobula in half along the dorso-ventral axis. (**B**) Number of VPN centroids in the dorsal and ventral lobula hemisphere. Some VPN populations show biases. (**C, D**) Same as in (A, B) but for the posterior (cyan) and anterior (purple) lobula hemispheres.

bioRxiv preprint doi: https://doi.org/10.1101/2024.04.24.591016; this version posted April 28, 2024. The copyright holder for this preprint (which was not certified by peer review) is the author/funder, who has granted bioRxiv a license to display the preprint in perpetuity. It is made available under aCC-BY-NC-ND 4.0 International license.

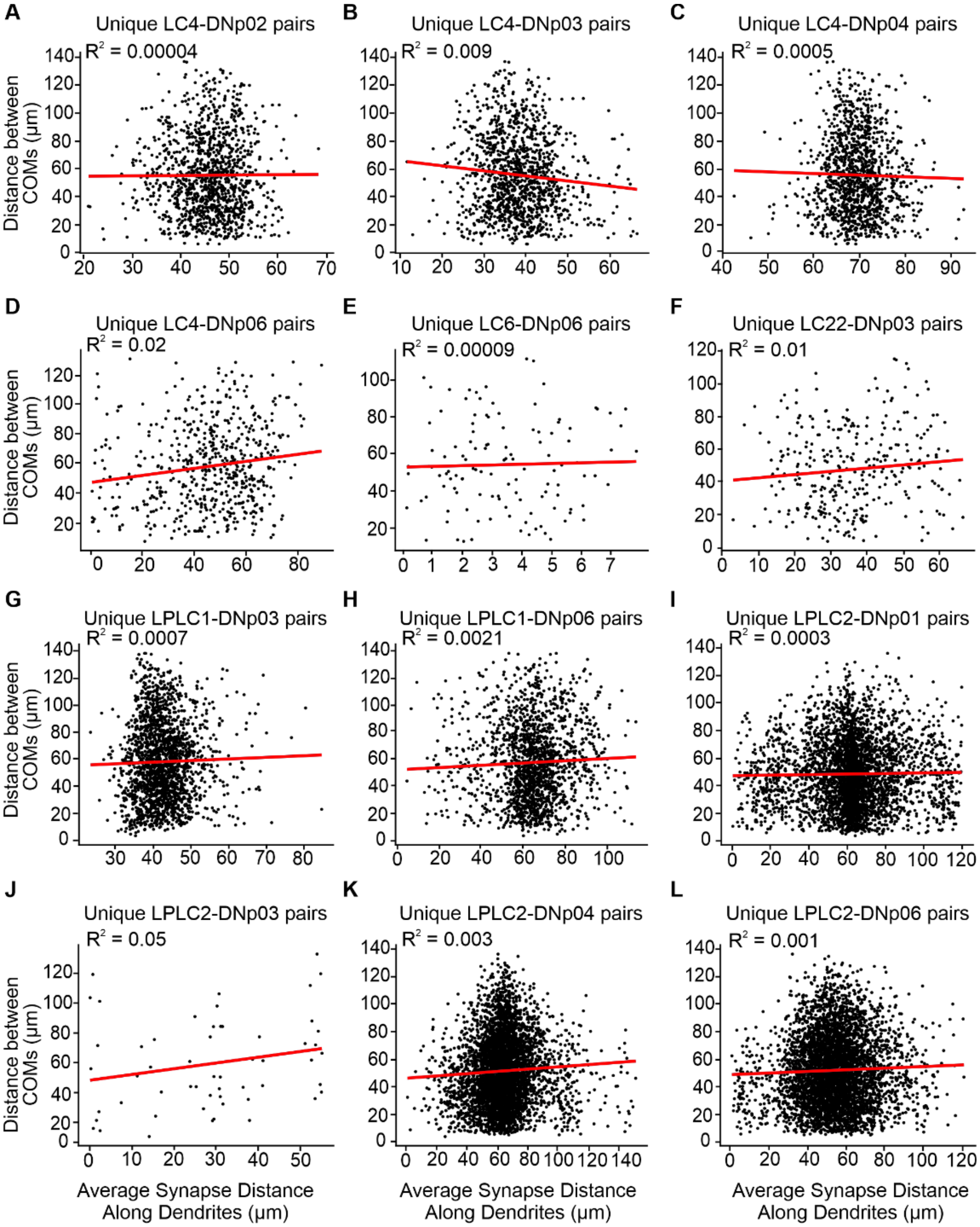

Figure 4—figure supplement 1. Lack of retinotopic synapse organization in all VPN-DN pairs. Linear regression line in red. **(A-L)** (**A**) The distance between the centroids of all unique pairs of LC4 presynaptic to DNp02 is not correlated with the average synapse distance for that given pair of LC4 neurons. (**B**) Same as (A) but for LC4-DNp03. (**C**) Same as (A) but for LC4-DNp04 (**D**) Same as (A) but for LC4-DNp06 (**E**) Same as (A) but for LC6-DNp06 (**F**) Same as (A) but for LC22-DNp03 (**G**) Same as (A) but for LPLC1-DNp03 (**H**) Same as (A) but for LPLC1-DNp06 (**I**) Same as (A) but for LPLC2-DNp01 (**J**) Same as (A) but for LPLC2-DNp03 (**K**) Same as (A) but for LPLC2-DNp04 (**L**) Same as (A) but for LPLC2-DNp06

bioRxiv preprint doi: https://doi.org/10.1101/2024.04.24.591016; this version posted April 28, 2024. The copyright holder for this preprint (which was not certified by peer review) is the author/funder, who has granted bioRxiv a license to display the preprint in perpetuity. It is made available under aCC-BY-NC-ND 4.0 International license.

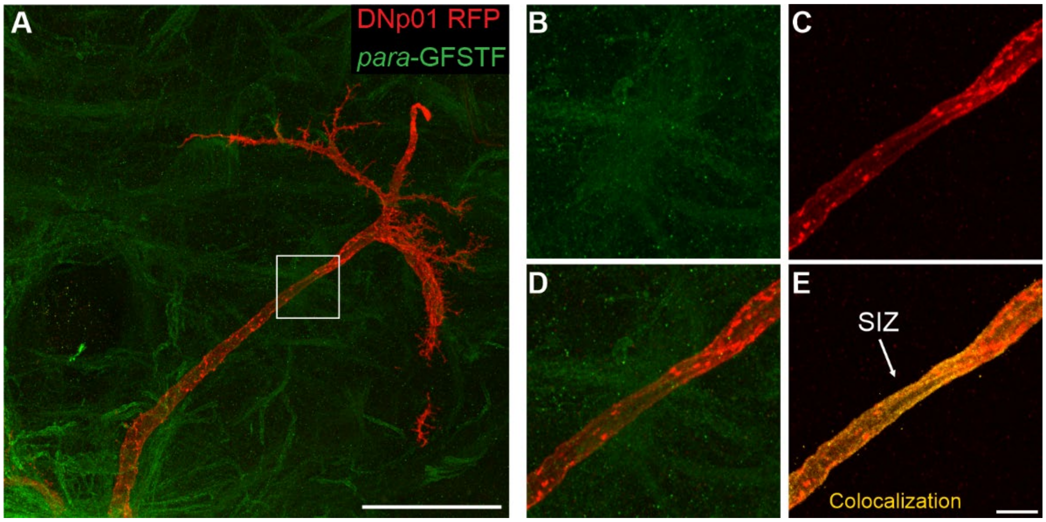

Figure 8—figure supplement 1. The SIZ is located downstream of the tether in DNp01. (**A**) Maximum intensity projection of RFP labeled DNp01 and GFP labeled para (genotype: *para-GFSTF, DNp01-split-GAL4, UAS-RFP).* Scale bar: 50 µm. Crosses were set at 22°C. (**B-E**) Zoomed-in view of (A) showing (**B**) the individual para and (**C**) DNp01 channels, (**D**) their overlay, and (**E**) para colocalization on DNp01 Scale bar: 5 µm.

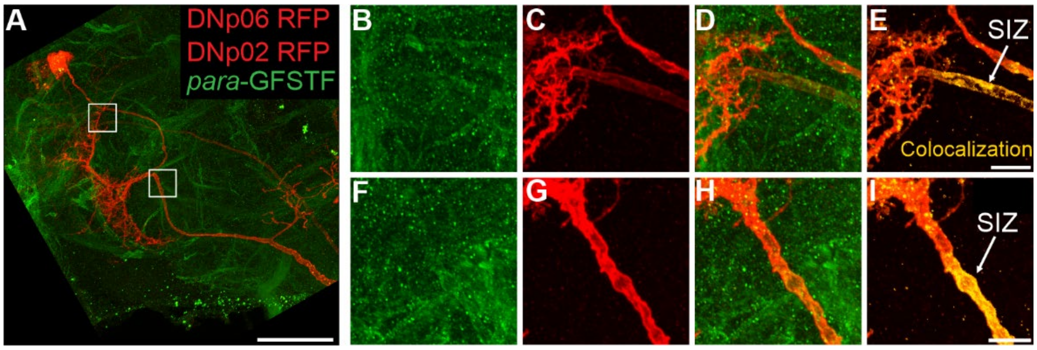

Figure 8—figure supplement 2. The SIZ is located downstream of the tether in DNp02 and DNp06. (**A**) Maximum intensity projection of RFP labeled DNp06 and DNp02 and GFP labeled para (genotype: *para-GFSTF, DNp06-split-GAL4, UAS-RFP*). Scale bar: 50 µm. Crosses were set at 18°C. (**B-E**) Zoomed-in view of top white box in (A) showing (**B**) the individual para and (**C**) DNp06/DNp02 channels, (**D**) their overlay, and (**E**) para colocalization on DNp06. Scale bar: 5 µm. (**F-I**) Zoomed-in view of bottom white box in (A) showing (**F**) the individual para and (**G**) DNp06/DNp02 channels, (**H**) their overlay, and (**I**) para colocalization on DNp02. Scale bar: 5 µm.

bioRxiv preprint doi: https://doi.org/10.1101/2024.04.24.591016; this version posted April 28, 2024. The copyright holder for this preprint (which was not certified by peer review) is the author/funder, who has granted bioRxiv a license to display the preprint in perpetuity. It is made available under aCC-BY-NC-ND 4.0 International license.

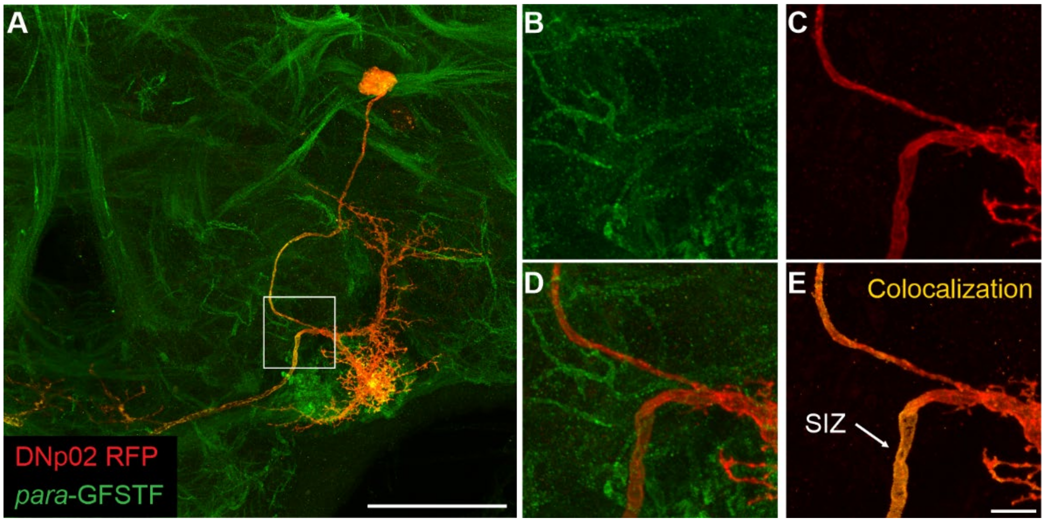

Figure 8—figure supplement 3. The SIZ is located downstream of the tether in DNp02. (**A**) Maximum intensity projection of RFP labeled DNp02 and GFP labeled para (genotype: *para-GFSTF, DNp02-split-GAL4, UAS-RFP).* Scale bar: 50 µm. Crosses were set at 18°C. (**B-E**) Zoomed-in view of (A) showing (**B**) the individual para and (**C**) DNp02 channels, (**D**) their overlay, and (**E**) para colocalization on DNp02 Scale bar: 5 µm.

bioRxiv preprint doi: https://doi.org/10.1101/2024.04.24.591016; this version posted April 28, 2024. The copyright holder for this preprint (which was not certified by peer review) is the author/funder, who has granted bioRxiv a license to display the preprint in perpetuity. It is made available under aCC-BY-NC-ND 4.0 International license.

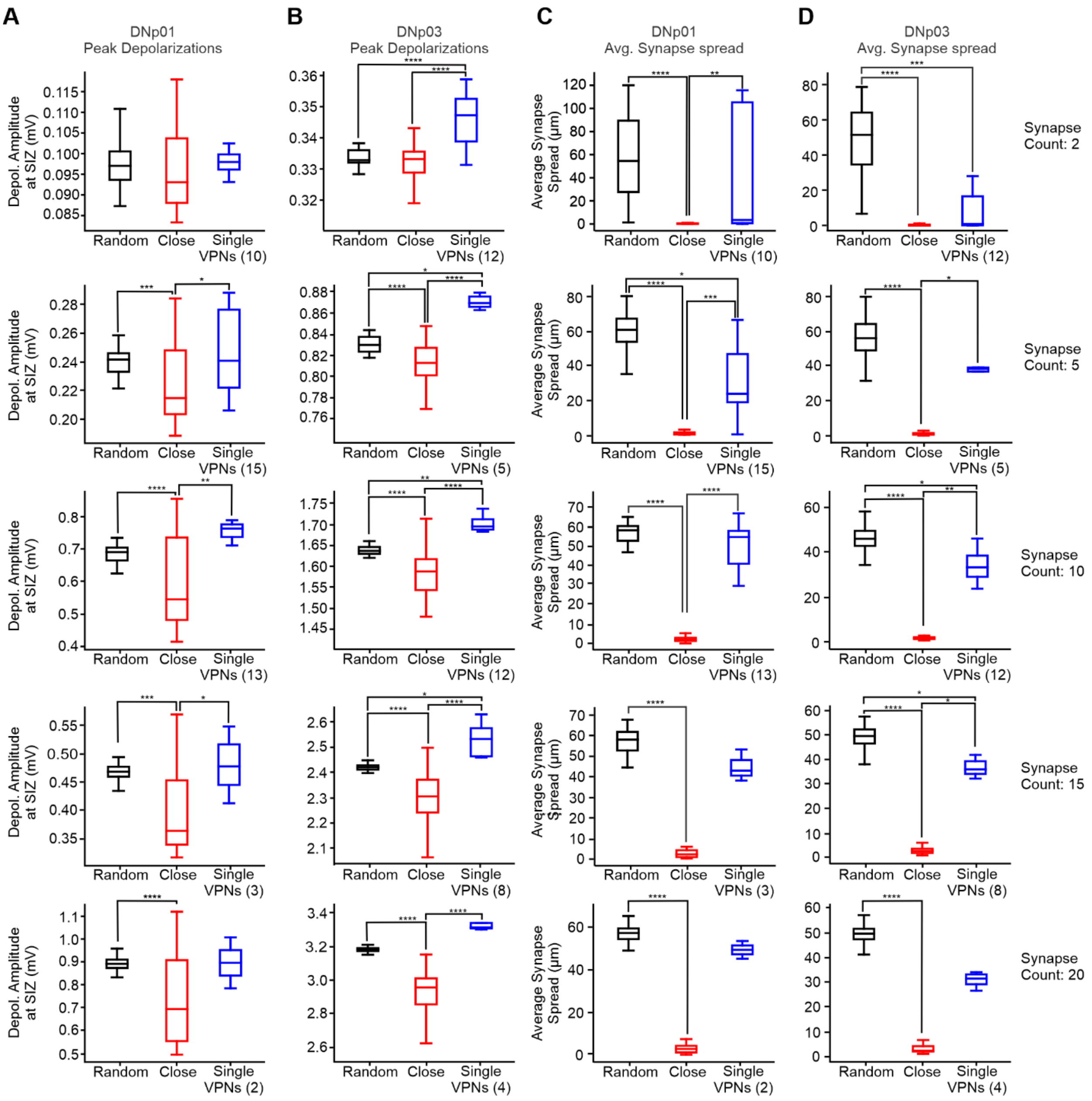

Figure 12—figure supplement 1: VPNs distribute their synapses to achieve efficient composite EPSP amplitudes at the SIZ. **(A, B)** Composite EPSP amplitudes at the SIZ in response to simultaneous activation of varying numbers of synapses in DNp01 (A) and DNp03 (B). **(C,D)** Average synapse spread of randomly distributed synapses, closely clustered and single VPNs at varying synapse counts in DNp01 (A) and DNp03 (B).
